# Thinking outside tuberculosis: A case of widespread active disseminated cysticercosis

**DOI:** 10.4102/sajr.v28i1.3016

**Published:** 2024-11-29

**Authors:** Audrey R. Rumhumha, Suraya Arbee, Mapule P. Mlawuli, Megan van Gensen

**Affiliations:** 1 Department of Diagnostic Radiology, Faculty of Health Sciences, University of the Witwatersrand, Johannesburg, South Africa

**Keywords:** active disseminated cysticercosis, disseminated tuberculosis, multiorgan parasitic infection, breast cysticercosis, neurocysticercosis, radiological investigations, immunocompromised, antihelminthics

## Abstract

A rare case of widespread disseminated cysticercosis was identified in a 32-year-old female patient from the Eastern Cape province, South Africa. Initially, she displayed generalised nonspecific symptoms, leading to a misinterpretation of disseminated tuberculosis (TB). However, further radiological, haematological and pathological investigations revealed extensive dissemination of cysticercosis.

**Contribution:**

Disseminated cysticercosis (DC) is a rare condition that can mimic symptoms of other diseases, including tuberculosis, highlighting the importance of considering it in endemic areas.

## Introduction

Cysticercosis is a systemic parasitic infestation caused by the pork tapeworm, *Taenia solium*.^1^ The parasite is transmitted to humans through the consumption of raw, fresh food, contaminated with tapeworm eggs.^2^ It is highly prevalent in rural areas of developing countries with poor sanitary conditions, and it is now recognised globally as one of the most prevalent food-borne parasitic diseases.^3^ The primary sites of manifestation include the central nervous system (CNS) (in spaces such as the subarachnoid area, ventricles, or spinal cord), subcutaneous tissues, lungs, eyes, liver, skeletal muscles and, sporadically, the pancreas, thyroid and heart.^4^ Disseminated cysticercosis (DC) represents an uncommon variant resulting from the dissemination of the larval phase of the pork tapeworm. The diagnosis of DC is confirmed by the presence of multiple vesicular cystic lesions in the brain, along with cystic presentations in at least two additional body sites.^5^ Widespread dissemination of the cysticercal infestation can result in the involvement of any organ in the body. Less than 50 cases of DC have been documented globally, with the majority of cases from India.^6^

## Ethical considerations

Ethical clearance to conduct this study was obtained from the University of the Witwatersrand, Human Research Ethics Committee (reference no: M240671). Patient consent was obtained for the case presentation, and the full anonymity of the patient has been maintained throughout the study.

## Patient presentation

### Clinical presentation

A 32-year-old immunocompromised female, non-vegetarian, presented with a 2-month history of nonspecific abdominal and generalised complaints, including loss of weight, constipation and abdominal pain. On the day of presentation, the patient complained of a week of constipation, nausea and vomiting for 3 days, and abdominal cramps. On physical examination, she was clinically stable with normal vital signs.

#### Radiological investigations

**FIGURE 1:**
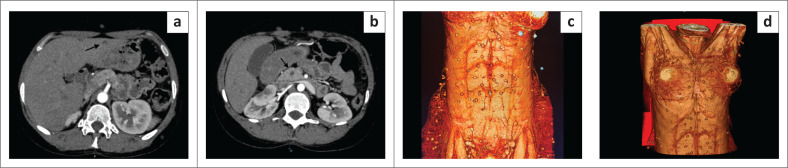
Axial CT abdomen showing rounded scattered cystic lesions within the liver (a), pancreas (b), and 3D reformatted image of the trunk demonstrating widespread subcutaneous nodules (c) and (d).

**FIGURE 2:**
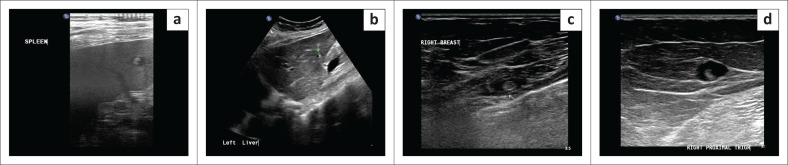
Lesions in the spleen (a) and liver (b) exhibiting a cystic appearance with a central area of increased echogenicity (representative of a scolex). Subcutaneous thin-walled cystic lesion with a central echogenicity (scolex) in right breast (c) and right anterolateral proximal thigh region (d).

**FIGURE 3:**
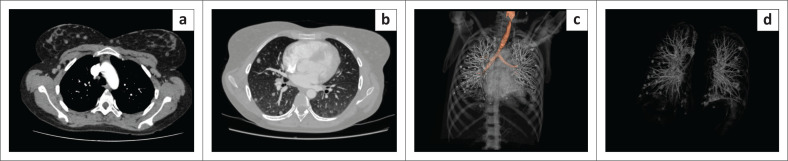
Axial CT chest images (a) and (b) and volume rendered (VR) reconstructed images (nodule analysis application) of the chest (c) and lungs (d) display numerous well-defined nodular lesions bilaterally as well as in the overlying chest wall tissues and breasts.

An initial clinical impression of possible bowel obstruction was made, and a CT scan of the abdomen was performed. Imaging revealed multiple hypodense focal lesions in the liver and spleen and innumerable subcutaneous nodules (Figure 1). Distended loops of large bowel were present without mechanical obstruction. Additional nodules were seen within the visualised lung bases. Imaging differentials included abdominal tuberculosis (TB) with hepatic and splenic microabscesses, suspected pulmonary metastatic lesions and potential hydatid disease. The cutaneous nodules were thought to possibly represent a phakomatosis. However, this was deemed to be less likely due to the absence of prior clinical history suggestive of this condition. The patient was admitted to a medical ward for further investigation as intestinal obstruction was excluded. Abdominal ultrasound indicated numerous round to oval, thin-walled, fluid-filled anechoic lesions with a focal central echogenic spot, indicating scolices in the spleen and liver. (Figure 2). A CT scan of the chest confirmed the presence of innumerable lung parenchymal nodules (Figure 3).

**FIGURE 4:**
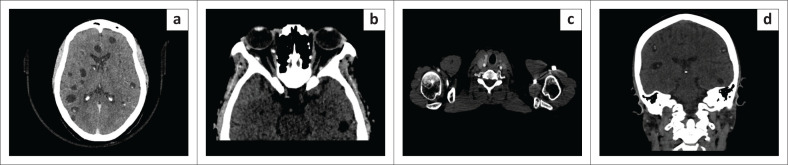
Axial CT brain (a) with the classic appearance of thin-walled cystic lesions with central hyperdensity representing a scolex, as well as focal round calcifications. Other sites of the head and neck manifestations of the disease are demonstrated with further classic appearing thin-walled cystic lesions with central hyperdensity representing a scolex on axial CT brain (b) within the retroocular structures (medial and lateral oblique muscles), axial neck CT (c) within the thyroid gland and on the coronal image (d) within both parotid glands.

A biopsy of the subcutaneous lesions was recommended and subsequently performed. At the time of biopsy, similar lesions were identified on ultrasound throughout the subcutaneous tissues and superficial muscles of the chest, abdomen, back, buttocks, lower limbs (Figure 2) and within the breast tissues. Further testing included a CT scan of the brain, which demonstrated multiple thin-walled cystic lesions, with the largest measuring 1.7 cm × 1.5 cm, scattered throughout the brain parenchyma. These had a hyperdense central dot indicating a scolex, consistent with the vesicular stage of neurocysticercosis, as well as some scattered, round, sub-centimetre calcifications, consistent with the nodular calcified stage. Additional manifestations of the disease affecting the head and neck were present within the retroocular structures, thyroid gland and bilateral parotid glands (Figure 4).

#### Haematological investigations

These were conducted alongside the radiological evaluations during the admission. Full blood count showed a decreased haemoglobin level of 8.8 g/dL, a reduced leukocyte count of 3.68 × 10^9^/L, and thrombocytosis of 560 × 10^9^/L. Eosinophilia was noted in the differential leukocyte count at 10.10%, and the C-reactive protein (CRP) level was elevated at 130, suggestive of a parasitic infection. The HIV test was positive, with a CD4 count of 104 cells/UL. The hepatitis screen was negative for hepatitis B surface antigen. Liver function tests displayed elevated total protein at 105, low albumin at 34 g/L, decreased total bilirubin at 4 nmol/L, reduced direct bilirubin at 3 nmol/L, normal alanine transaminase (ALT) at 20 U/L, normal aspartate transaminase (AST) at 19 U/L, high alkaline phosphatase (ALP) at 106 U/L, and elevated gamma-glutamyl transferase (GGT) at 49 U/L.

Lumbar puncture was performed, and analysis revealed clear, colourless fluid with normal glucose levels at 2.9 mmol/L, protein levels at 0.32 g/L, and adenosine deaminase (ADA) levels at 0.6 U/L. Lymphocytes were elevated at 20 U/L, and no polymorphs or erythrocytes were observed. The venereal disease research laboratory (VDRL) test was negative, and tests for India ink-encapsulated yeast, gram stain bacteria, and cryptococcal antigen all returned negative results. Bacterial culture showed no growth after 2 days. All TB investigations were negative, including urinary lipoarabinomannan (LAM) and sputum GeneXpert analysis.

#### Pathological investigations

A core biopsy of the right breast lesion demonstrated adipose and fibrous connective tissue and an inflammatory reaction of lymphocytes, plasma cells, and eosinophils, confirming a helminthic infection with no evidence of invasive malignancy. It was concluded that the biopsy contained only a fragment of the organism precluding full visualisation, but findings aligned closely with cysticercosis, based on the clinical context and radiological evidence. Additionally, fine needle aspiration (FNA) was performed on a cervical lymph node, which revealed blood and inflammation with tingible body macrophages, consistent with a benign process.

The diagnosis of DC was established following comprehensive clinical, imaging, and pathological investigations. The patient was commenced on a treatment regimen of albendazole 500 mg twice daily, praziquantel 3.5 g daily, and dexamethasone, with significant clinical improvement after 2 weeks of treatment.

## Discussion

Disseminated cysticercosis is a rare and challenging manifestation of cysticercosis involving the widespread dissemination of cysticerci throughout the body. It can, thus, manifest with a myriad of symptoms depending on the organs involved. The diagnosis of cysticercosis poses significant challenges due to its nonspecific clinical manifestations, occasionally non-pathognomonic imaging findings, and the low sensitivity and specificity of serological tests. Del Brutto et al. established diagnostic criteria that encompass clinical, imaging, immunologic and epidemiological data, which are classified into absolute, major and minor features to facilitate the categorisation of diagnoses as either definite or probable.^7^ Imaging thus plays a pivotal role in the diagnosis and management of DC. CT and MRI are the mainstay modalities for evaluating CNS involvement, while ultrasonography and CT are preferred for extra-neural manifestations.^1^ A comprehensive imaging evaluation is necessary to identify the extent of dissemination, as was demonstrated in the presented case. Ancillary tests such as serological assays and molecular techniques may complement imaging studies in equivocal cases.^6^

Radiologic findings in cysticercosis vary based on the type of cysticercus, the larval development stage, and cyst location and quantity. Neuroimaging reveals four distinct stages of cyst formation. The vesicular stage appears as a hypodense cyst with a hyperintense scolex and a nonenhancing or mildly enhancing wall on CT. In the colloidal vesicular stage, there is the formation of a fibrous capsule and surrounding oedema, characterised by ring-enhancing cystic lesions on CT. The granular nodular stage features a retracted cyst forming an enhancing nodule with mild oedema. Finally, in the calcified stage, the lesion is shrunken and fully calcified, presenting as one or more calcified nodules on CT.^4^ MRI is the preferred imaging modality for diagnosing neurocysticercosis due to its superior resolution and absence of ionising radiation.^1^ Additionally, radiographs may reveal ‘rice grain’ calcifications within muscles during the calcified stage of the disease. In the presented case, the criteria for a definite diagnosis were satisfied, with one absolute feature (a cystic lesion with a scolex on CT), two minor features (clinical manifestations and cysticercosis outside the CNS), and an epidemiological feature (immigration from an endemic area). While the results of the breast biopsy further corroborated our diagnosis, they were not essential, as the definite diagnostic criteria had already been fulfilled.

The majority of patients with DC typically exhibit neurocysticercosis as the primary manifestation,^8,9^ with the second most prevalent site being myocysticercosis.^10^ What made the patient in this study unique was her presentation showcasing extensive multiorgan involvement, including the brain, orbits, thyroid, parotids, breasts, lungs, liver, pancreas, spleen, muscle and subcutaneous tissues. Despite conducting a thorough literature review, there was a scarcity of cases involving breast and pulmonary organs, with none showcasing cysticerci in the liver.

Cysticercosis in the breast remains a rare occurrence, with the breast considered an atypical site for this condition.^11^At a leading medical institution in India, only eight cases of cysticercosis were identified among 8364 breast aspirates.^8^ A broader examination over 21 years revealed a total of 28 cases of parasitic infections in the breast, including 16 instances of cysticercosis and 12 of filariasis, as confirmed through fine needle aspiration cytology (FNAC). Similarly, a study conducted in Nepal analysed 23 402 biopsy specimens of suspected cysticercosis from various sites such as skin nodules, oral mucosa, and breast tissue. Of these, there were 62 documented cases of cysticercosis, with 8% of those cases being found in the breast.^12^

A case report by Bhalla et al. highlighted the potential for atypical presentations of this condition with involvement of the spleen and pancreas in DC,^6^ emphasising the diverse clinical spectrum of this condition. A case of pulmonary involvement was reported by Savigamin et al.,^2^ with a patient presenting with lung nodules. The importance of considering DC in the differential diagnosis of patients presenting with multisystem involvement, especially in endemic regions, is emphasised by Kumar et al.^4^ In typical cases, cysticercosis manifests as calcifications resembling rice grains within the soft tissues; however, in this instance, diffuse cystic lesions were present. The thyroid and parotid glands also represent uncommon sites for cysticercosis,^13^ with the authors’ literature review revealing only a limited number of reported cases of thyroid and parotid involvement, typically associated with DC rather than isolated occurrences.

The patient in this study, who was immunocompromised and due to non-adherence to antiretroviral therapy, presented with a low CD4 count of 104 cells/UL. While the precise role of immune function in the presentation of cysticercosis remains poorly defined in humans, it is accepted that a compromised immune system increases susceptibility to widespread infections.^14^ Research conducted in Mexico demonstrated an association between immunodeficiency and an elevated incidence of neurocysticercosis among paediatric patients. There have been documented cases of individuals with immunocompromising conditions, such as leukaemia, exhibiting widespread but asymptomatic cysticercosis, supporting the association of an impaired immune system on the disease’s presentation.^14,15^

Due to the varied clinical presentations, a multidisciplinary approach involving neurologists, radiologists and infectious disease specialists is important in the accurate diagnosis and management of DC. The management of DC involves a multimodal approach focused on controlling symptoms, eliminating viable parasites and preventing disease recurrence. Antiparasitic medications like albendazole and praziquantel are central to treatment, often used alongside corticosteroids to reduce inflammation.

## Conclusion

Widespread DC is a rare and complex form of cysticercosis, marked by the widespread distribution of cysticerci throughout the body. Recent studies have shed light on its varied clinical manifestations, diagnostic challenges and therapeutic approaches, highlighting the need for a thorough understanding of DC for early diagnosis and effective intervention. Further research is essential to clarify its pathogenesis, improve diagnostic methods and enhance treatment strategies to reduce the disease burden in endemic areas.
